# Examining Ankle Foot Orthosis Wear Time in Patients With Peripheral Artery Disease

**DOI:** 10.1093/geroni/igaa057.682

**Published:** 2020-12-16

**Authors:** Sara Myers, Danae Dinkel, Mahdi Hassan, Holly Despiegelaere, Jason Johanning, Iraklis Pipinos

**Affiliations:** 1 University of Nebraska at Omaha, Omaha, Nebraska, United States; 2 University of Nebraska Omaha, Omaha, Nebraska, United States; 3 VA Medical Center, Omaha, United States; 4 University of Nebraska Medical Center, Omaha, Nebraska, United States

## Abstract

Peripheral artery disease (PAD) impacts over 8.5 million Americans and the prevalence of PAD increases with age. PAD restricts blood flow to the leg and its most common manifestation is claudication, a severe impairment of walking produced by ischemia-related, leg pain during exercise. An ankle foot orthosis (AFO) could improve these symptoms. To understand the potential impact of AFO usage, it is critical to determine wearability of the device in patients with PAD. The purpose of this study was to monitor wear time of an AFO and explore perceptions of the device. Participants (n=14) with PAD and claudication wore an AFO for three months. An accelerometer was placed directly on the AFO for 7 days and participants completed semi-structured interviews at midpoint (1.5 months) and post intervention (3 months). Based on accelerometer data at midpoint participants wore the AFO for an average of 4.9±2.3 out of 7 days and for an average of 7.5±4.2 hours each day. At post, participants wore the AFO for an average of 4.8±2.2 days for an average of 7.4±4.6 hours per day. In the interviews, almost all participants noted multiple barriers to wearing the AFO such as difficulty putting the AFO on and off, using stairs, walking on uneven ground, and driving. Our study found that participants wore the AFO ~7 hours/day but experienced barriers which may have limited their wear outside of these monitoring periods suggesting patients would wear an assistive device if design could be improved to address barriers.

